# Editorial: Antiplatelet Agents in Stroke Prevention

**DOI:** 10.3389/fneur.2021.762060

**Published:** 2021-09-28

**Authors:** Gergely Feher, David Hargroves, Zsolt Illes, Peter Klivenyi, Liping Liu, Laszlo Szapary

**Affiliations:** ^1^Centre for Occupational Medicine, Medical School, University of Pécs, Pécs, Hungary; ^2^East Kent Hospitals University NHS Foundation Trust, Ashford, United Kingdom; ^3^Department of Neurology, Odense University Hospital, Odense, Denmark; ^4^Department of Neurology, Faculty of Medicine, Albert Szent-Györgyi Clinical Center, University of Szeged, Szeged, Hungary; ^5^Beijing Tiantan Hospital, Capital Medical University, Beijing, China; ^6^Medical School, University of Pécs, Pécs, Hungary

**Keywords:** stroke, antiplatelet agent, resistance, biomarker, outcome

Stroke is the leading cause of disability and the second most common cause of death worldwide based on the results of the Global Burden of Diseases Study (1). More than 80% of all stroke syndromes are ischemic infarcts and their prevalence and cost will undoubtedly rise as aging populations increase (2). Despite extensive risk factor stratification and enhanced brain imaging, the etiology of stroke is still unknown in a significant proportion of patients. However, atherosclerosis, which is a low-grade inflammatory condition with detectable biomarkers, is the most likely culprit in most strokes (3).

Platelets play an essential role in the pathogenesis of atherothrombotic cardio- and cerebrovascular events, thus justifying the use of antiplatelet agents in their prevention. In their mini review, Valis and his workgroup summarized the evidence-based role of antiplatelet agents in the secondary prevention of non-cardioembolic stroke including aspirin, clopidogrel, dual antiplatelet therapy, and alternative agents such as cilostazol and ticagrelor Vališ et al.

Despite their efficacy, patients on these medications continue to suffer complications, which raises the possibility of the so-called “antiplatelet resistance” that is used to refer to the inability to protect individuals from thrombotic events (4). Due to the lack of standard methodology and randomized trials involving cerebrovascular patients, the clinical significance of antiplatelet resistance is contradictory (5). However, observational studies have shown an increased rate of ischemic cerebrovascular events in patients with high on-treatment of platelet reactivity (HPR) (so called resistance) in patients with both single (SAPT) and dual antiplatelet therapy (DAPT) (6).

Kang et al. analyzed the risk factors of clopidogrel resistance in patients taking mono- and dual therapy Kang et al. They demonstrated that HPR is more frequent in recurrent stroke patients receiving clopidogrel SAPT than in those receiving DAPT, and its risk factors may differ. The rates of HPR and clopidogrel resistance were lower in current smokers, which is rather surprising as smoking is one of the most important risk factors of atherosclerotic diseases. The role of smokers' paradox is not well-understood and merits further investigation.

In their paper Schrick et al. presented a modified platelet function test (mPFT) wherein they not only tested whole blood (WB), but also analyzed 1-h gravity sedimentation of the separated upper (UB) and lower half blood (LB) samples using Multiplate Analyzer to detect HPR as well as neutrophil antisedimentation rate (NAR) Shrick et al. This pilot study suggested that upward motion of platelets might be associated with increased thrombotic tendency.

It is worth noting that assessment of response to aspirin, GPI-s, or PAR-inhibitors is clinically not established as suggested by the Working Group on Thrombosis of the European Society of Cardiology (7). The most reliable, clinically best validated, and most widely used assays measured the effect of P2Y12-inhibitors (clopidogrel or prasugrel) and the recommended techniques were VASP-P assay, the VerifyNow device, and the Multiplate analyzer (7, 8). However, the routine use of platelet function testing is still not recommended (7, 8).

HPR can be associated with more ischemic events and recent studies have shown an increased bleeding risk in patients with low platelet reactivity (LPR) (9). Rosafio et al. also presented an interesting clinical case scenario of an aspirin ultra-responder patient Rosafio et al.

Since the coagulation system plays an important role in stroke pathogenesis, blood biomarkers of coagulation, and inflammation might render the possibility to differentiate which patients are at risk of poor clinical outcome. The ability to predict clinical outcome after an ischemic stroke may help to improve the selection of the most appropriate therapy (10). Based on recent studies, hemostatic changes during acute stroke in relation to antiplatelet resistance may predict the severity of an ischemic stroke.

In their in-depth review, Alhazzani et al. summarized the integration of specific biomarkers, genotype-, as well as phenotype-related data in antiplatelet therapy stratification in patients with acute ischemic stroke, which could be of great clinical impact on outcome Alhazzani et al.

Platelet endothelial aggregation receptor-1 (PEAR1) rs12041331 has been reported to affect agonist-stimulated platelet aggregation which can be associated with HPR in aspirin and clopidogrel treated patients, increasing the risk of unfavorable outcome. An observational Chinese study conducted by Zhang et al. could not confirm its role either in ischemic nor in bleeding events in TIA or minor stroke patients taking DAPT, doubting its prognostic value Zhang et al.

There is no doubt that taking antiplatelet agents or anticoagulants increases the risk of bleeding complications. Antiplatelet (especially DAPT) pretreatment potentially increases the risk of intracranial bleeding in thrombolyis/thrombectomy situations as well as in patients with traumatic brain injuries (11, 12). The potential harmful effects of DAPT have also been confirmed in this issue by the research of Lin et al. in more than 1,000 elderly patients with moderate to severe strokes who underwent systemic thrombolysis Lin et al. Although the patient cohorts were quite homogenous, the DAPT group contained relatively few patients (~2% of the study cohort). Finally, based on a recent meta-analysis consisting of more than 60,000 patients, DAPT did not appear to be associated with a higher risk of adverse outcomes in thrombolyzed stroke patients, so dual pre-treatment is not an indication to withdraw treatment, which is also confirmed by the authors (13).

Single small subcortical infarction (SSSI or lacunar stroke) accounts for 25% of all strokes and has heterogenous pathogenesis. Recent studies have shown an increased bleeding risk of SSSI patients, especially for those with underlying small vessel disease or taking DAPT (14). As the optimal treatment of these patients is not entirely clarified, Wang et al. analyzed the data of the CHANCE trial dividing patients into different subgroups based on antiplatelet treatment and SSSI etiology Wang et al. They could not find any differences in the outcome of different subgroups, which merits further investigation.

Endovascular treatments have recently proven to be effective in improving functional outcomes for selected patients with large vessel occlusion, although it can cause injury to endothelial cells leading to activation of local platelet aggregation and subsequent early reocclusion, and therefore more effective and safe thrombolytic agents are required (15). Glycoprotein (GP) IIb-IIIa inhibitors are short-acting selective reversible antiplatelet agents widely used in acute coronary syndromes and have recently emerged as promising therapeutic agents for ischemic stroke management. Among them, tirofiban may be considered safe in low doses (15). Two studies focused on the efficacy and safety of tirofiban in relation to the management of large vessel occlusion (LVO) including thrombectomy. Huo et al. showed its beneficial effects in 650 ischemic stroke patients; based on their findings tirofiban was found to be associated with superior clinical outcomes in anterior circulation stroke and major stroke patients and had a trend to lower the risk of mortality at 90-day follow-ups with no increase in bleeding rates compared to the non-tirofiban group Huo et al. In the other study presented by Ma et al. covering ~200 patients, no significant differences in safety and efficacy outcomes on successful recanalization, clinical improvement, or 3-month mRS could be found between the tirofiban and non-tirofiban groups Ma et al. The administration of tirofiban seems to be safe in LVO patients but its efficacy and safety merits further investigation.

Intracerebral hemorrhage (ICH) may be caused by antiplatelet treatment and prior treatment may be associated with worse clinical outcomes; however, previous studies on ICH growth and outcome have found conflicting results (16, 17). In their meta-analysis of 31 studies, Wu et al. found no association with hematoma expansion or functional outcomes in ICH patients, but increased mortality rates raised the possibility of the introduction of early-time platelet function reversal strategies Wu et al. It is worth noting that the randomized PATCH trial found platelet transfusion to be inferior compared to standard care in ICH patients (18).

The rupture of an intracranial aneurysm could be a life-threatening disease accounting for a relatively small but significant number of stroke syndromes. The role of prior antiplatelet use on the risk of bleeding and outcome is not well-studied. In their interesting meta-analysis covering nearly 9,000 participants, Yang et al. found that prior aspirin use was associated with a significantly lower risk of aneurysm growth and rupture, suggesting the potential protective effect of aspirin Yang et al. However, it is not well-understood and merits further investigations.

## Author Contributions

This editorial was written by GF and checked by DH, ZI, PK, LL, and LS. All authors contributed to the article and approved the submitted version.

## Conflict of Interest

The authors declare that the research was conducted in the absence of any commercial or financial relationships that could be construed as a potential conflict of interest.

## Publisher's Note

All claims expressed in this article are solely those of the authors and do not necessarily represent those of their affiliated organizations, or those of the publisher, the editors and the reviewers. Any product that may be evaluated in this article, or claim that may be made by its manufacturer, is not guaranteed or endorsed by the publisher.
